# The emerging roles and therapeutic potential of exosomes in epithelial ovarian cancer

**DOI:** 10.1186/s12943-017-0659-y

**Published:** 2017-05-15

**Authors:** Xiaoduan Li, Xipeng Wang

**Affiliations:** Department of Gynecology, Shanghai First Maternity and Infant Hospital Affiliated with Tongji University, Shanghai, China

**Keywords:** Exosomes, Ovarian cancer, Biomarker, Therapy, Drug resistance

## Abstract

Ovarian cancer (OC) is one of the three types of malignant tumors in the female reproductive system, and epithelial ovarian cancer (EOC) is its most typical form. Due to the asymptomatic nature of the early stages and resistance to chemotherapy, EOC has both a poor prognosis and a high fatality rate. Current treatments for OC are very limited, and the 5-years survival rate is approximately 30%. Exosomes, which are microvesicles ranging from approximately 30–100 nm in size that are secreted by living cells, can be produced from different cell types and detected in various body fluids. Cancer cells can secrete more exosomes than healthy cells, and more importantly, the content of cancer cell-derived exosomes is distinct. The exosomes shedding from tumor cells are considered to be involved in tumor progression and metastasis. As such, exosomes are expected to be potential tools for tumor diagnosis and treatment. In this review, we briefly present the emerging roles of exosomes in OC and summarize related articles about their roles as diagnostic or prognostic biomarkers and in the treatment and drug resistance of OC.

## Background

Epithelial ovarian cancer (EOC) is one of the most malignant tumors in the female reproductive system. As cancer statistics in China [1] revealed, the mortality rate of ovarian cancer (OC) has been rising for the past few years (comparing 2001–2003 with 2003–2011). In addition, this trend also existed in the United States, and the estimated mortality rates of OC would rank as the fifth highest based on existing data (2007–2011) [2]. Unlike other tumors, OC tumors tend to metastasize within the peritoneum but rarely disseminate through the vasculature [3], suggesting that OC may require treatment that is different from that for other tumors. Current clinical treatments for OC are very limited, and the surgery (cytoreduction or debulking), chemotherapy or treatment with a novel agent are the main therapeutic options. More than 75% of women have advanced disease (stage III or IV) at the time of diagnosis, that the 5-years survival rate among these patients is less than 25% [4]. Due to the asymptomatic nature of this disease during the early stages and resistance to chemotherapy, a poor prognosis can be expected [5].

Exosomes were proposed as products of membrane exfoliation at first. Johnstone first described and named them “exosomes” in 1987, and he suggested that exosomes might have specific membrane-related functions [6]. The potential primary function of exosomes is intercellular communication. Exosomes may have distinct biological activities, and their components depend on the cellular origin and are highly variable [7]. Exosomes contain many proteins, mRNAs, miRNAs, DNAs, lipids and transcriptional factors. In addition, a multitude of pathways can be activated because of cellular interactions with exosomal molecules, including mRNAs, miRNAs and proteins (e.g., heat shock proteins [HSPs] and adhesion molecules) [8].

It has been gradually accepted that cancer cells secrete more exosomes than healthy cells. Moreover, the contents of these exosomes have been found distinct from each other [9]. Remarkably, cancer cell-derived exosomes can provide a suitable microenvironment for cancer development, such as cell proliferation [10–12], drug resistance [13], angiogenesis and metastasis [14], immune modulation [15] and premetastatic niche formation [16]. In addition, these features are all present in OC. Therefore, exosomes are expected to be potential diagnostic, prognostic, and even therapeutic tools for EOC. Certainly, such applications are still under development and far from clinical translation. Exciting research has shown that exosomes can serve as drug delivery systems, similar to nanoparticles. Scientists loaded exosomes with chemotherapeutic drugs, which can be used to kill tumor cells effectively without typical side effects [17]. Based on current nanoparticle research, such drug delivery systems will have more applicability in clinic.

Although the field of exosomes is relatively new, they are involved in OC and expected to have applications in OC as well. In this review, we briefly present the emerging roles of exosomes in OC and summarize related articles about their roles as diagnostic or prognostic biomarkers and in the treatment and drug resistance of OC, especially EOC.

## Exosomes as biomarkers of OC

The biomarker mainly used for the diagnosis and prognosis of EOC is CA125, which has low sensitivity and specificity. Recent studies have revealed that exosomes can transport enzymes from EOC cells. Furthermore, an increased level of exosomes in the peritoneal fluid has been found to be correlated with tumor progression [18, 19], indicating that the level of exosomes in the blood is correlated with the stage of OC. Thus, exosomes themselves and their contents have potential as tumor-specific markers. Although no exosomes-related indicator comparable with CA125 has been widely accepted. In addition, the lack of large-scale clinical trials and the immature purification and detection methods remain unresolved issues. The writers still believe that exosomes are promising as biomarkers of OC.

### Exosomal miRNAs as diagnostic and prognostic biomarkers

Recent studies have shown that the role of exosomes was protecting miRNAs from RNases, which created conditions for using exosomal miRNAs to diagnose OC [20]. MiRNA levels are important and often detected. Eight particular miRNAs (e.g., miR-21) in exosomes isolated from the serum of women with benign disease or various stages of EOC demonstrated diagnostic promise, because they were not detectable in the normal controls [21]. Those findings indicate that miRNAs of circulating tumor-derived exosomes could potentially be used as surrogate diagnostic markers for biopsy profiling or extended to screening asymptomatic populations. A similar phenomenon was observed in miR-222-3p, compared with healthy individuals, the expression of exosomal miR-222-3p was significantly increased in the serum of EOC patients [22]. MiR-21 was involved in the oncogenesis of ovarian serous carcinoma (OSC). The exosomal transfer of miR-21 could promote oncogenic transformation in target cells distant from the primary tumor, and it could be used as a diagnostic tool [23]. The high concentrations of exosomes in EOC patients suggested excessive and active exosomal secretion. In a cohort of 163 EOC patients, the levels of miR-200b and miR-200c were higher in International Federation of Gynecologists and Obstetricians (FIGO) stage III-IV patients, including those with lymph node metastasis, than in FIGO stage I-II patients [24]. This analysis showed that these miRNAs were highly suitable for tumor staging, diagnosis and prognosis.

Exosomes in the urine have gained attention, and urinary exosomal miRNAs are easily available samples that have been recently explored, particularly in urological and gynecological diseases. Zavesky confirmed a significant up-regulation of miR-92a in OC urinary samples, which could use for diagnostic purposes [25]. In addition, miRNA microarray data showed that miR-30a-5p was up-regulated in the urine samples of ovarian serous adenocarcinoma (OSA) patients compared with healthy controls. Furthermore, lower urine levels of miR-30a-5p were found in gastric cancer (GC) and colon carcinoma (CC) patients, suggesting that urinary miR-30a-5p may be particular to OC and demonstrating the use of exosomal urinary miR-30a-5p as a specific diagnostic marker [26]. However, the diagnostic potential of exosomal urinary miRNAs may depend on the urine fraction and might be vulnerable to the external environment, which is an obvious constraint.

Considering that the sources of exosomal miRNAs were very extensive, and differed from one another, the type and expression level of miRNAs was very different. Moreover, the various studies mentioned before had gathered information on patients from different type of ovarian cancer, thus they presented different characteristics in the number of patients, varied specimen and quantity. Despite all the research with one table list, it was lack of inconsistency between different studies.

### Exosome-related proteins as biomarkers

In this section, we summarize exosome-related proteins, such as endosomal membrane proteins, HSPs, enzymes, and globulins. These proteins are involved in adhesion, angiogenesis, proliferation, and metabolism [27]. In-depth proteomic analyses of OC cell line-derived exosomes revealed the differential enrichment of functional proteome categories [28]. When examining the protein profile of purified exosomes derived from two OC cell lines (OVCAR-3 and IGROV1), 1017 proteins were identified in both exosome types, including all the major exosomal protein markers. These data indicated that exosomes might serve as a resource of blood-based protein biomarkers in OC [29]. Comparing malignant and cirrhotic ascites, a proteomic analysis identified 424 proteins specific to malignant ascites. In addition, a functional analysis demonstrated that the major differences between them were the clusters of spliceosome proteins. The OC ascites proteome may provide markers for predicting patient survival and the effectiveness of different chemotherapy methods [30]. OC patient’s plasma contains higher levels of exosomal proteins than that of benign tumor patients or healthy controls. Significantly, high exosomal protein levels were observed in both newly diagnosed OC patients and those with advanced stages of OC. In addition, the levels varied when examined during and after chemotherapy [31]. As a result, changes in exosomal protein levels might be useful for predicting responses to therapy and prognosis in OC patients when combined with clinical data.

Epithelial cell adhesion molecule (EpCAM) overexpression correlated with an escalation in epithelial cell proliferation during tumor development [32]. The exosomal EpCAM levels in normal individuals were very low, while an increase in the exosomal EpCAM level was found to be related to the stage and severity of ovarian carcinoma. These findings indicated that EpCAM could be useful for the diagnosis of OC with the detection of tumor-derived exosomes [21]. CD24 is an established marker of poor prognosis in ovarian and other types of carcinomas, and it has been identified in exosomes isolated from the ascites fluid of ovarian carcinoma patients. What is more, most of the CD24-positive exosomes are secreted by tumor cells [33]. EpCAM and CD24 in ovarian tumor-derived exosomes are promising alternatives for early detection [34]. In addition, they can distinguish OC-derived exosomes from vesicles originating from normal cells. Furthermore, as shown in a small set of pre- and post-treatment OC patient ascites samples, changes in the exosomal EpCAM and CD24 levels can accurately classify patients as being responsive or nonresponsive to therapy [35, 36], which has great value for evaluating prognosis. Activated leukocyte cell adhesion molecule (ALCAM) is involved in cell-cell interactions in cancer. Serum ALCAM levels have also been found to be significantly higher in EOC patients than in controls, and full-length transmembrane ALCAM was detected in exosomes extracted from EOC ascites samples. Studies have shown that ALCAM is a marker of EOC and correlates with more aggressive tumors [37].

Tumor-reactive immunoglobulins can also be used as diagnostic markers in OC. Serum samples from OC patients exhibited significantly greater immunoreactivity levels than samples from either normal controls or women with benign disease. Analyzing IgG recognition of specific exosomal antigens can distinguish benign ovarian masses from OC, as well as early- and late-stage OC. The quantitative assessment of IgG reactivity with tumor-derived exosomal proteins can be used as diagnostic markers for OC [38]. In addition, claudin proteins frequently overexpressed in OCs, and full-length claudins can be found in OC cell-derived exosomes. Moreover, nearly half of the plasma samples from OC patients exhibited the presence of claudin-4-containing exosomes [39]. Thus, claudin-4 can be used for the detection of OC.

A proteomics analysis of exosomes derived from two late-stage OC cell lines demonstrated that the pentose phosphate pathway was a dominant mechanism in exosome-mediated intracellular communication. Glucose-6-phosphate dehydrogenase (G6PD), transketolase (TK) and transaldolase 1 (TA1) are three key enzymes in the regulation of this pathway and may become diagnostic or prognostic biomarkers of OC [40]. The inhibition of N-glycosylation processing caused changes in the composition of extracellular vesicles and induced a decrease in the levels of several glycoproteins, which showed that the identified glycosignatures of extracellular vesicles could serve as novel biomarkers for OC [41].

Yong Zeng et al. found that the ExoSearch chip offers a method for the isolation and release of blood/plasma exosomes in various preparation volumes (10 μL to 10 mL). In addition, the blood-based diagnosis of OC via three exosomal protein markers for tumor (CA125, EpCAM, and CD24) showed significant diagnostic power [42] in that tumor testing with a drop of blood was possible. All biomarkers related to exosomes are summarized in Table 1.

**Table 1:** Exosomes serve as biomarkers of OC

Exo-related	Content	Effect	Research subjects	Refer.
MiRNA	miR-222-3p	M2 polarization diagnosis	EOC patients healthy individuals	[22]
	miR-21	oncogenesis diagnostic tool	OSC cases and cystadenoma and normal ovaries	[23]
	miR-373 miR-200a miR-200b miR-200c	diagnosis prognosis	EOC patients healthy women	[24]
	miR-92a	diagnosis	OC patient urine samples	[25]
	miR-30a-5p	diagnosis (high specificity)	OSA patient urine samples GC and CC patients	[26]
Proteins	1017 proteins	protein biomarkers	OVCAR-3 and IGROV1 cells	[29]
	cluster of spliceosomal proteins	prognostic markers	OC ascites and cirrhotic ascites	[30]
	EpCAM	diagnosis prognosis	women with malignant and benign ovarian disease OC patients	[21] [36]
	CD24	early detection prognosis	ovarian carcinoma ascites OC patients	[33] [36]
	ALCAM	aggression biomarker	EOC ascites	[37]
	IgG	diagnosis	women with OC and benign ovarian	[38]
	claudin-4	detection	OC patients and healthy volunteers	[39]
	enzymes (G6PD, TK, TA1)	diagnosis prognosis	OVCA429 and HO8910PM ovarian cell lines	[40]
	glycoproteins	biomarker	ovarian carcinoma OVM cells	[41]

## Exosomes as prospctive therapeutic targets in OC

### Exosomes are related to the immune system, which may provide the foundation for future immunotherapies

The limitations of current chemotherapies for OC have motivated the development of new treatments. Evidence has shown that exosomes released by a tumor can suppress the immune system of a patient and prepare niches for metastatic spread [43]. Cancer-related exosomes exhibit powerful influences that benefit tumors via a variety of biological mechanisms. Understanding the underlying and complex exosome-mediated immunosuppressive mechanisms is important to prevent the immune escape of tumor cells and identify novel treatments for cancer. The ability to block immunosuppressive factors in tumor microenvironments is expected to enhance antitumor immune responses in OC patients.

Myeloid-derived suppressor cells (MDSCs), whose main feature is the potent immunosuppressive effect, are a major component of the tumor microenvironment. MDSCs are a heterogeneous group that includes macrophages, tumor-associated macrophages (TAMs), dendritic cells (DCs), monocytes, and granulocytes [44]. Dendritic and lymphoid exosomes can regulate immune activation. Tumors can release exosomes mimicking membranous material, resulting in the deletion of reactive lymphocytes. In addition, these exosomes expressed many reactive molecules, which suppressed the expression of T cell activation signal components, thus suppressing the immune activity of T cells and inducing apoptosis [45]. Exosomes isolated from the malignant ascites of OC patients could be internalized by monocytes and induced the production of cytokines. Exosomes from ascites can trigger toll-like receptor (TLR)-dependent signaling pathways in monocyte-precursor cells, which are important for the induction of immunosuppressive mechanisms during cancer progression [46]. The function and differentiation of macrophages can also be adjusted by exosomes. EOC-derived exosomes can activate macrophages to exhibit a TAM-like phenotype, which can facilitate the progression of cancer. MiR-222-3p can transfer from EOC cells to macrophages by exosomes, thereby effectively regulating the polarization of tumor-promoting M2 macrophages [22]. In addition, EOC exosomes have shown to be involved in the immunosuppression of natural killer (NK) cells, which are confirmed to be important in two cytotoxic pathways for anticancer immunity, the NK C-type lectin-like receptor (NKG2D) receptor-ligand pathway and the CD226/DNAM-1-polio virus receptor/nectin-2 pathway [47].

In addition, exosomes isolated from malignant ascites can also impair the cytotoxic activity of peripheral blood mononuclear cells (PBMCs), and Fas ligand (FasL) has shown to be present in the exosomal suspension. Thus, anti-FasL antibody can decrease the percentage of DC and PBMC apoptosis [48]. The Fas/FasL system plays an important role in the immune privilege status of tumors by inducing Fas-mediated apoptosis in tumor-specific lymphocytes. Normal ovarian surface epithelial cells express but do not secret FasL, while EOC cells secrete FasL via the release of exosomes. The release of secreted FasL rather than the membrane-bound form facilitated the escape from immune surveillance and survival of the tumor cells [49]. Lysophosphatidic acid (LPA) can up-regulate FasL presentation on the surface of OC cells and stimulate the secretion of FasL-bearing exosomes, thereby contributing to an OC cell counterattack against activated T cells and promoting OC metastasis [50]. Ovarian carcinoma-derived exosomes have shown to carry phosphatidylserine (PS) on their surface, and PS appeared to be an important molecule for exosomal uptake by NK cells [51]. The PS present on the membrane of exosomes isolated from ovarian tumor tissues or ascites can reversibly inhibit the activation of T cells by arresting the T cell signaling cascade. The depletion or blockade of PS with anti-PS antibody could significantly block the inhibition of T cell function, which represents a potential therapeutic approach for patients with OC [52].

Scientists have leveraged the relation of exosomes to the immune system in antitumor research since 2005. One clinical trial employed an immunotherapeutic approach in conjunction with conventional first-line chemotherapy. The immunotherapy based on the use of a TLR3 agonist and tumor-derived exosomes carrying associated antigens, which could disrupt tumor immune tolerance and immunosuppressive effects. This therapy also exploited tumor-derived exosomes as a potential source of antigens for generating effective and lasting tumor antigen-specific T cell immunity [53]. In another study, metastatic ovarian carcinoma-derived exosomes presented tumor-specific antigens to DCs derived from umbilical cord blood, and these DCs could then stimulate common T cells to differentiate and effectively induce cytotoxicity, thereby providing a novel immunotherapy for OC [54] The potential targets for immunotherapy are listed in Table 2. Although there are currently few applications, the authors believe that the role of exosomes in tumor microenvironment immunity may have the most potential for treating OC.

**Table 2:** Exosomes provide potential targets for immunotherapy

Exo-resource	Target cells	Effect	Potential therapeutic targets	Refer.
EOC cells	macrophages	M2 polarization	miR-222-3p	[22]
Malignant ascites	monocytes	cytokine production	TLR-dependent signaling pathways	[46]
EOC cells	NK cells	cytotoxicity inhibition	NKG2D and DNAM-1 ligands	[47]
Malignant ascites	PBMCs and DCs	apoptosis induction	FasL	[48]
OC cells	T cells	counterattack against activated T cells	LPA and FasL	[50]
Ovarian tumor tissues	T cells	T cell inhibition	depletion or blockade of PS	[52]

### Exosomes are related to the tumor microenvironment and promote progression and metastasis

OC patients have a poor prognosis and frequently experience recurrence and dissemination, even with chemotherapy or surgical debulking. Recent evidence revealed that the tumor microenvironment consists of stromal cells (including fibroblasts, macrophages, and MDSCs) and extracellular matrix components (including inflammatory cytokines and chemokine), which can promote cancer cell invasion and metastasis [55]. An increasing amount of evidence has shown that exosome-mediated interactions between tumor cells and their microenvironment are a key factor in tumor progression. Exosomes are key mediators of intercellular communication in the tumor microenvironment [56]. Intercellular communication is a function that progressively developed via a process of mutual cellular adaptation. Experiments showed that when cancer cells co-cultured with different individual mesenchymal stem cells (MSCs), the interactions between the MSCs and cancer cells depended on the exchange of exosomes. During these cellular interactions, varieties of functional changes were observed, particularly those promoting the growth of tumor cells [57].

As previously mentioned, urinary exosomal miR-30a-5p could be used as a high-specificity diagnostic marker of OC. At the same time, miR-30a-5p knockdown significantly inhibited OC cell proliferation and migration. Therefore, miR-30a-5p can also serve as a therapeutic target for OSA [26]. It has been found that exosomes, which present in the ascites and blood of ovarian carcinoma patients, contain different exosomal proteins frequently related to tumor progression, such as metalloproteinase inducers (EMMPRIN/CD147) and pro-heparanase. The application of patient-derived exosomes can promote the growth of human SKOV3ip carcinoma xenografts in mice [51]. Although this was just a preliminary animal experiment, the results suggested that the application of exosomes in the treatment of OC was feasible.

Exosome shedding, which is frequently observed in tumor cells, is suggested to be involved in several aspects of tumor progression. It demonstrated that exosomes isolated from invasive tumor cell lines as well as the bodily fluids of OC patients can deliver membrane-type 1 matrix metalloprotease (MT1-MMP), which is involved in matrix degradation and disease progression [58]. Extracellular matrix degradation played an important role in OC metastasis. In OC, ascites-derived exosomes were found to contain gelatin lysis enzymes. In addition, exosome-associated proteolytic activity in the tumor vicinity might augment tumor cell invasion into the stroma. Fractionation of malignant ascites revealed exosomes containing localized extracellular matrix-degrading proteinases. The activation of proteinases, such as MMPs and urokinase-type plasminogen activator (uPA), led to increased extracellular matrix degradation, which may facilitate tumor cell invasion and metastasis [59]. Human OC cell lines (CABA I and A2780) shed exosomes carrying MMPs into the extracellular medium, demonstrating a mechanism for regulating focalized proteolytic activity and interacting with the microenvironment [60]. The L1 adhesion molecule (CD171) overexpressed in EOC and associated with a poor prognosis. Although expressed as a transmembrane molecule, L1 was released from carcinoma cells in a soluble form. Tumor-derived vesicles may be an important source of soluble L1 that can regulate tumor cell function in an autocrine-paracrine fashion. Soluble L1 from ascites showed to be a potent inducer of cell migration [61].

More researches are required to provide insight into the mechanisms underlying exosome-induced invasion and migration. Recent studies revealed that LIN28, an RNA-binding protein, could be highly expressed in the exosomes of OC cells (IGROV1) and be internalized by non-tumor cells. When taken up by HEK293 cells, these exosomes led to significant increases in the expression of genes involved in the epithelial-to-mesenchymal transition (EMT) and induced HEK293 cell invasion and migration [62]. The exosomal release of various miRNAs significantly correlated to the invasive potential of cancer cells, and some miRNAs showed significant associations with effusion site and tumor stage. For example, high miR-21, miR-23b and miR-29a levels were associated with poor progression-free survival, whereas high miR-21 expression was correlated to poor overall survival [63]. OC cells specifically secrete exosomes with miRNAs. Then, these exosomes communicate with mesothelial cells to allow tumor dissemination through the peritoneal cavity. When comparing OC cell lines with high (Skov-3) and low (OVCaR-3) invasive potential, transcripts of the miR-let-7 family were more abundant in OVCaR-3 cells than Skov-3 cells. In contrast, they were more abundant in exosomes from Skov-3 cells than OVCaR-3 cells. Transcripts from the miR-200 family was only identified in OVCaR-3 cells and their exosomes [64]. These findings might because miR-let-7 suppresses cell proliferation by transporting more exosomes outside of cells, while miR-200 suppresses the EMT.

Malignant ascites is a natural medium for cell communication. A cluster of spliceosomal proteins identified in ascites as prognostic markers for the prediction of patient survival. Furthermore, they played a role in communication between cancer cells [30]. As previously mentioned, the pentose phosphate pathway is a dominant mechanism mediating intracellular communication via exosomes excreted from OC cells. Therefore, G6PD, TK, and TA1 are key proteins that could become therapeutic targets of OC [40].

A novel concept for the treatment of OC based on impairing the crosstalk between metastatic ovarian cells and their environment has been tested. And a tumor cell capture device was fabricated with exosomes and a three-dimensional scaffold (metastatic trap [M-Trap]). M-Trap transformed a systemic disease into a focalized disease, in which proven therapeutic approaches, such as surgery, can extend survival [65]. Certainly, there are exosomes that directly possess antitumor effects, such as disintegrin and metalloproteinase 15 (ADAM15)-rich exosomes. ADAM15 can be released into the extracellular space as a component cleaved from released exosomes, and this effectively inhibited cancer cell migration and activation [66]. In another study, exosomes from MSCs inhibited cell cycle progression in all tested cell lines and induced Skov-3 cell necrosis. An increase in the number of cells in the G0-G1 phase was observed, suggesting a block in cell cycle progression. These results indicated that exosomes could halt proliferation, thus leading to cell death by apoptosis or necrosis [67]. The functions of exosomes described previously are showed in Table 3.

**Table 3:** Exosomes related to tumor progression, metastasis and angiogenesis

Exo-function	Exo-resource	Mechanism	Potential therapeutic targets	Refer.
Proliferation	OSA	miR-30a-5p is an oncogenic miRNA	miR-30a-5p	[26]
	malignant ascites	exosomes contain tumor progression-related proteins	EMMPRIN/CD147 pro-heparanase	[51]
Invasion/Migration/Metastasis	Invasive tumor cell lines. bodily fluids of OC patients	VAMP3 regulates the delivery MT1-MMP in exosomes	MT1-MMP	[58]
	malignant ovarian ascites	increased extracellular matrix degradation	MMPs and uPA	[59]
	OC cell lines (CABA I and A2780)	proteolytic activity	MMPs	[60]
	ovarian carcinoma cell lines malignant ascites	L1 is cleaved by ADAM10	L1 adhesion molecule (CD171)	[61]
	OC cell line (IGROV1)	increases genes involved in the EMT	LIN28	[62]
	OC patients	educate mesothelial cells to allow dissemination	miRNAs in exosomes	[63]
	OC cell lines (Skov-3 and OVCaR-3)	miR-let-7 suppresses cell proliferation miR-200 suppresses the EMT	miR-let-7 miR-200	[64]
Intracellular communication	OC and cirrhotic ascites	signal transduction	cluster of spliceosomal proteins and RNAs	[65]
	ovarian cell lines (OVCA429 and HO8910PM)	pentose phosphate pathway	G6PD, TK, TA1	[40]
Angiogenesis	OC cell lines	affect VEGF or HIF-1α	ATF2 and MTA1	[27]
	ovarian carcinoma cells	CD147 stimulates VEGF expression	CD147	[68]
	SKOV3 cells	down-regulate IGF1R	miR-375	[69]

### Exosomes are related to angiogenesis

To date, curative therapies for OC remain under development, and anti-angiogenesis approaches may provide alternative strategies. Tumor-derived exosomes are essential for tumor angiogenesis.

High-grade OC-derived exosomes have a more profound impact on angiogenesis than those derived from low-grade OC. Proteomic profiles revealed that the constituents of high-grade OC-derived exosomes, such as activating transcription factor 2 (ATF2) and metastasis-associated protein 1 (MTA1), among others, had a profound impact on angiogenesis and may play a key role in enhancing tumor development [27]. CD147 is a membrane molecule that is highly expressed in tumor cells and is involved in the progression of malignancies by regulating MMP expression in peritumoral stromal cells. A recent study showed that CD147 expressed in exosomes derived from EOC cells and that CD147-positive vesicles shed by OC cells may induce angiogenesis activities in human umbilical vein endothelial cells [68].

Anti-angiogenesis effects may serve as the mechanisms of some chemotherapy drugs and be expected to provide targets for the treatment of OC. Recently, a study demonstrated the anti-neoplastic effect of Amla extract (*Emblica officinalis*, AE) on OC in vitro and in vivo. The underlying mechanism was that AE could down-regulate pro-angiogenic molecules, such as insulin-like growth factor 1 receptor (IGF1R), via up-regulating cellular and exosomal miR-375 in human OC cells [69], explaining why AE attenuated angiogenesis and had an antitumor effect.

## Exosomes are related to drug resisitance

There is no doubt that OC is the most lethal gynecological malignancy. Most OC patients are initially sensitively responsive to the preferred chemotherapy with platinum and paclitaxel (PTX). Unfortunately, most patients experience recurrence within 6–12 months and die of progressively chemotherapy-resistant disease [70].

One study showed that cancer-associated adipocytes (CAAs) or fibroblasts (CAFs) can secrete exosomes to transfer miR-21 into neighboring cancer cells, thereby increasing the chemo-resistance of these cells to PTX via the down-regulation of the direct target apoptotic protease-activating factor 1 (APAF1). Exosomal miR-21 can enhance OC cell PTX-resistance and decrease OC cell apoptosis [71]. The relationship between endogenous miR-433 expression and resistance to PTX was also been investigated in OC cell lines. Moreover, miR-433 mediated the down-regulation of cyclin-dependent kinase 6 (CDK6) [72]. Interestingly, miR-21-3p induced cisplatin (CIS) resistance in ovarian tumors, potentially by targeting the neuron navigator 3 (NAV3) gene. Exosomes released by CIS-resistant cells were also capable of increasing resistance in other cells [73].

In addition to miRNAs, exosomal proteins are also involved in the process of tumor drug resistance. After being shed, exosomes can transfer their contents to recipient cells at the membrane-bound protein level. For example, P-glycoprotein (P-gp) shared between human OC cells via intercellular exosomal transfer, resulting in enhanced cellular resistance to PTX [74]. Jie Y et al. found that increased annexin A3 expression was a mechanism for platinum resistance in OC cells [75]. Further research showed that serum annexin A3 levels were significantly higher in platinum-resistant patients than in platinum-sensitive patients. It was also found that annexin A3 was associated with exosomal release from platinum-resistant OC cells, which was supported by OC cells expressing higher levels of annexin A3 releasing increased numbers of exosomes [76]. Proteins may be transports or carriers of chemotherapy drugs. The lysosomal proteins ATPase copper-transporting alpha and beta (ATP7A and ATP7B), which are putative CIS-export transporters, can enhance the exosomal export of platinum in drug-resistant human ovarian carcinoma cells [77].

Now, the supporting role on drug resistance of exosomes sourced from tumor microenvironment has, gain more and more attention, such as exosomes from CAAs or CAFs. Although there are few related research in ovarian cancer, we believe that they will increase rapidly, thus we still listed them separately in Table 4.

**Table 4:** Exosomes related to drug resistance

Exo-source	Specific source	Content	Drugs	Target/Mechanism	Refer.
cancer-related cells	CAAs or CAFs	miR21	paclitaxel	APAF1	[71]
cancer cells	A2780 cells	miR-433	paclitaxel	CDK6	[72]
	A2780 (PTX/WT) cells	P-gp	paclitaxel	export chemotherapeutic agents	[74]
	A2780 and CP70 cells	miR-21-3p	carboplatin	NAV3 gene	[73]
	sera from OC patients SKOV3/CIS cells	annexin A3	carboplatin	prevent uptake or accumulation of platinum in cells	[76]
	CIS-resistant cells	ATP7A, ATP7B	carboplatin	CIS-export transporters	[77]

## Conclusions

Due to the asymptomatic nature of this disease during the early stages and resistance to chemotherapy, OC, particularly EOC, has a poor prognosis [5]. The distinct components and biological activities of exosomes must be explored to identify novel high-sensitivity and high-specificity biomarkers for the early diagnosis of EOC. Such exosomal biomarkers should significantly increase in OC patients compared with healthy individuals, which are higher in patients with advanced-stage disease than early-stage disease. Some miRNAs meet these requirements, such as miR-222-3p [22], miR-200b and miR-200c [24]. However, as previously mentioned, related therapies remain in the laboratory stage and there is a lack of large-scale clinical trials. In addition, the diagnostic potential of any biomarker depends on the sample type (e.g., serum and urine, among others), and the maturity of the purification and detection methods will be a constraint. The limitations of current clinical OC therapies require the development of new treatments. Cancer-related exosomes exhibit powerful influences on tumors by a variety of biological mechanisms, and it is important to understand the underlying mechanisms of exosome-mediated immunosuppression and tumor progression, metastasis and angiogenesis. The ability of exosomes block factors in tumor microenvironments should enhance antitumor responses in OC. Most EOC patients died of progressively chemotherapy-resistant disease notably [70]. In addition to miRNAs, exosomal proteins are also involved in the process of drug resistance in OC patients. In order to retain patient sensitivity to platinum and PTX, the mechanism of drug resistance should be more thoroughly understood, and exosomes provide a novel perspective in that context.

**Fig. 1:**
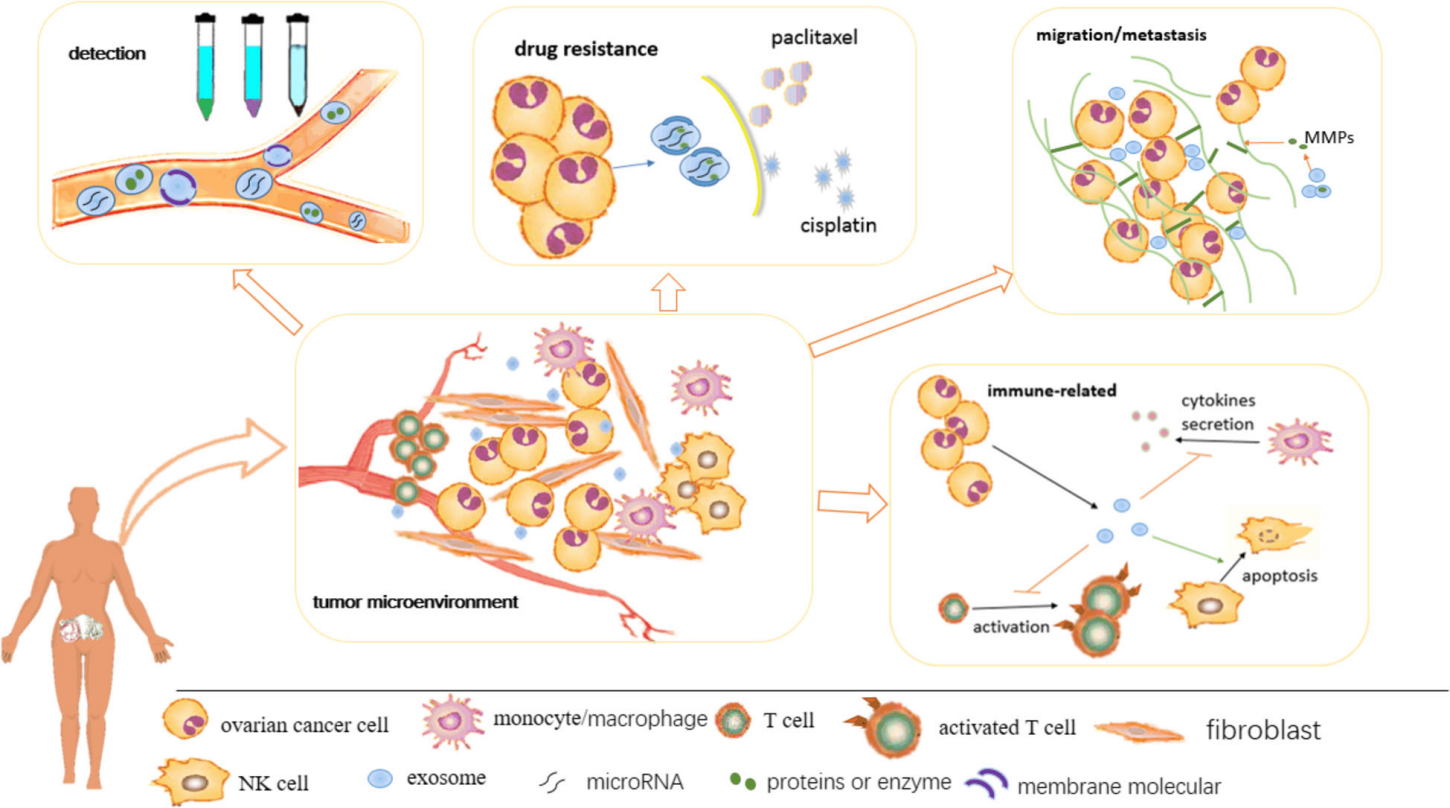
A summary of the roles of exosomes in ovarian cancer

In summary, the emerging field of exosomes has created new perspectives on diagnosis, prognosis, treatment, and drug resistance in OC (Fig. 1). Exosomes represent a mobile source of information regarding the molecular makeup of parental tumors. However, the exosomal knowledgebase has thus far been limited to the findings of a few persuasive studies. In addition, high-throughput exosomal isolation and analysis remains difficult. Much more research on exosomes needs to be perform to reveal breakthroughs in diagnosing and treating OC.

## Abbreviations

ADAM: a disintegrin and metalloproteinase
ALCAM: Activated leukocyte cell adhesion molecule
APAF1: Apoptotic protease-activating factor 1
ATF2: Activating transcription factor 2
ATP7A: ATPase copper-transporting alpha
ATP7B: ATPase copper-transporting beta
CAAs: Cancer-associated adipocytes
CAFs: Cancer-associated fibroblasts
CC: Colon carcinoma
CDK6: Cyclin-dependent kinase 6
CIS: Cisplatin
DCs: Dendritic cells
DNAM-1: CD226
EMMPRIN: Metalloproteinase-inducer
EMT: Epithelial-to-mesenchymal transition
EpCAM: Epithelial cell adhesion molecule
FasL: Fas ligand
G6PD: Glucose-6-phosphate dehydrogenase
GC: Gastric cancer
HIF-1α: Hypoxia-inducible factor-1 alpha
IGF1R: Insulin-like growth factor 1 receptor
LPA: Lysophosphatidic acid
M2: Macrophage 2 phenotypes
MDSCs: Myeloid-derived suppressor cells
MSCs: Mesenchymal stem cells
MT1-MMP: Membrane-type 1 matrix metalloprotease
MTA1: Metastasis-associated protein 1
NAV3: Neuron navigator 3
NK: Natural killer
NKG2D: NK C-type lectin-like receptor
OC: Ovarian cancer
OSA: Ovarian serous adenocarcinoma
OSC: Ovarian serous carcinoma
PBMCs: Peripheral blood mononuclear cells
P-gp: P-glycoprotein
PS: Phosphatidylserine
PTX: Paclitaxel
TA1: Transaldolase 1
TK: Transketolase
TLR: Toll-like receptor
uPA: urokinase-type plasminogen activator
VAMP3: Vesicle-associated membrane protein 3
VEGF: Vascular endothelial growth factor
WT: Wild type
